# Analysis of undiagnosed tuberculosis-related deaths identified at post-mortem among HIV-infected patients in Russia: a descriptive study

**DOI:** 10.1186/1471-2334-11-276

**Published:** 2011-10-18

**Authors:** Yanina Balabanova, Vladimir Tchernyshev, Igor Tsigankov, Svetlana Maximova, Natalya Mikheeva, Ljudmila Fedyukovitch, Sergey Kuznetsov, Ivan Fedorin, Francis Drobniewski

**Affiliations:** 1 National Mycobacterium Reference Laboratory, Center for Infectious Diseases, Institute of Cell and Molecular Sciences, Queen Mary College, Barts and the London School of Medicine, University of London, 2 Newark street, E1 2AT, London, UK; 2 Samara Oblast Tuberculosis Dispensary, 154 Novosadovaya str, 443068 Samara, Russia; 3 Togliatti City Tuberculosis Dispensary, 34 Telegraphnaya str, 445013 Togliatti, Russia; 4 Samara Oblast Minsitry of Health and Social Development, 44 Revolyutsionnaya str, 443054, Samara, Russia

## Abstract

**Background:**

Tuberculosis remains a serious public health threat and economic burden in Russia with escalating rates of drug resistance against a background of growing HIV-epidemic. Samara Oblast is one of the regions of the Russian Federation where more than 1% of the population is affected by the HIV-epidemic; almost half of the cases are concentrated in the largely-industrial city of Togliatti with a population of 800 000.

**Methods:**

We conducted a retrospective analysis of errors leading to death of HIV-positive patients in general health care hospitals in Togliatti, Russia, in 2008. All (n = 29) cases when tuberculosis was established at autopsy as a cause of death were included.

**Results:**

Median length of hospital stay was 20 days; in 11 cases the death occurred within the first 24 hours of admission. All cases were known to be HIV-positive prior to admission, however HAART was not initiated for any case, and no relevant tests to assess severity of immunosupression were performed despite their availability. No appropriate diagnostic algorithms were applied to confirm tuberculosis. Major gaps were identified in the work of hospital and consulting physicians including insufficient records keeping. In almost all patients earlier regular HIV-relevant tests were not performed due to poor compliance of patients, many of whom abused alcohol and drugs.

**Conclusions:**

We conclude that introduction of prompt and accurate diagnostics tests, adequate treatment protocols and intensive training of physicians in management of AIDS and TB is vital. This should include reviewing standards of care for HIV-positive individuals with accompanying social problems.

## Background

Tuberculosis (TB) remains a serious public health threat and economic burden in the Russian Federation with escalating rates of multi- and extensive drug resistance (MDR and XDR) against a background of poor infection control and active spread of resistance strains [1,2]. At the end of the 1990s, the HIV-epidemic emerged and is now spreading fast across the country with the number of registered cases being close to half a million by the end of 2009 [3]; the number of HIV-TB co-infection cases is increasing and according to national data TB is one of the most common causes of death of HIV-infected patients [4,5]. Due to the recent nature of the epidemic and the poor availability of antiretroviral drugs until recent years [6], Russian physicians have relatively limited experience of the diagnosis and treatment of HIV/AIDS cases and opportunistic infections [7-9]

Samara Oblast is one of the regions of the Russian Federation where more than 1% of the population is affected by the HIV-epidemic (over 42 000 registered cases by end-2010; population 3.3 million) [10]. Almost half of the cases are concentrated in the largely-industrial city of Togliatti with a population of 800 000. TB incidence and mortality rates among HIV-positive patients there are several-fold higher than among the general population (602.0/100 000 and 48.6/100 000 versus 83.4/100 00 and 9.5/100 000 respectively) [11].

We aimed to describe the characteristics of a series of TB-related deaths among HIV-positive patients admitted to general health care (GHC) hospitals in Togliatti, in 2008, with a diagnosis of TB established by post-mortem autopsy analysis (undiagnosed while the patients were alive). The study was initiated by Togliatti lead TB physicians with the objective of identifying diagnostic errors that had led to misdiagnosis of TB.

## Methods

### Study Settings

All general health care (GHC) hospitals in Togliatti.

### Study Population

All cases (n = 29) of death occurring in HIV-positive patients, in 2008, admitted via the emergency room into GHC hospitals in Togliatti with TB established as a cause of death at autopsy.

### Data Collection and Diagnostic Procedures

We collected medical data from each participant's case histories and autopsy reports. As the study was performed retrospectively, no additional interviews of relatives, physicians or further laboratory examinations were conducted. All presented information reflects the total data available and includes data on age at time of death, date of hospital admission, in-patient medical diagnosis at time of death, stage of the HIV infection, current HIV treatment history, history of TB in the past, and autopsy results.

Informed consent for autopsy was obtained from patients' relatives in all cases at the time of death. A limited autopsy was performed on all cases according to standard protocols for establishing the cause of death of all patients deceased at hospital settings [12]. Representative samples of internal organs were fixed in 10% formalin solution, in order to preserve the tissue for histological examination. Detailed description of all changes found macroscopically and microscopically was documented. Prior to microscopy, all tissue sections were stained routinely with haematoxylin and eosin. For each major organ, a maximum of three sections were taken. To detect acid-fast bacilli a section from every lobe of the lungs, one from the left and right main bronchus and trachea, the hilar and mediastinal lymph nodes was taken. Fresh gloves and blades were used for every organ sampled. Tissue sections were treated with special stains including Ziehl-Neelsen for acid-fast bacilli, Gram's stain for bacteria, periodic acid-Schiff for fungi and Giemsa stain for *Pneumocystis jeroveci*. In 100% of cases TB was established as the cause of death.

### Statistical Analysis

A descriptive analysis was conducted identifying the main characteristics of patients recruited into the study and the causes of death as reflected in the case histories.

### Ethical Approval

The study was approved by the Samara Ministry of Health and Social Development Ethics Review Board and received a waiver of informed consent due to the retrospective nature of the study and absence of additional procedures or additionally collected data.

## Results

All patients included in the analysis were admitted to GHC hospitals as emergency cases. They were known to be HIV-positive before hospital admission and clinical notes available at admission reflected that all were registered as patients at the city AIDS Center; all presented with advanced stages of infection (B2-B3 according to the CDC classification) [13].

The median length of hospital stay prior to death was 20 days (range 1 hour to 91 days); however in 11 cases death occurred within the first 24 hours of admission.

The majority of cases were males (n = 20; 69%). The median age was 32 years (range of 23-51 years). Most patients belonged to a socially disadvantaged group and 86% of them (n = 25) were unemployed. The vast majority (n = 26; 90%) had reported alcohol and drug abuse. All patients who reported drug-abuse also had accompanying hepatitis B and/or C infection.

Clinically, at admission the vast majority of patients presented with fever, fatigue, cough, and alcohol or drug intoxication (Table 1).

**Table 1:** Clinical symptoms of patients at admission to the hospitals (n = 29)

*Symptom*	*N*	*%*
Cough	23	79
Haemoptysis	2	7
Shortness of breath	8	29
Night sweats	26	90
Chest pain	5	17
Fatigue	29	100
Sudden weight loss	24	83
Fever	29	100
Alcohol intoxication symptoms	17	59
Drug intoxication symptoms	8	28

Although all patients were known to be HIV-positive, in none of the cases during the three months prior to hospital admission was a CD4 count or viral load (VL) test performed (usually due to the patients missing their appointment; these tests are free of charge for all patients). At admission, the CD4 count was performed in 7 (24%) patients and VL level was measured in one patient only. In cases when death occurred within the first 24 hours of admission failure to run these tests was explained by the very short time between admission and death while in other cases no reasons were provided. In all seven tested cases the CD4 count was significantly below 200 cells/ml^3^. None of the patients received highly active antiretroviral therapy (HAART) prior to admission.

In all cases a chest X-Ray (CXR) was performed, however, no abnormalities consistent with TB were reported initially. In twelve cases other abnormalities of an unidentified nature were reported. In these cases, a second routine review of the CXR was performed by radiologists who were not blinded towards the results of the first examination. It suggested that ten cases (35%) were presumptively TB arguing that there might have been an atypical presentation due to the advanced stage of immunosuppression. In two of these patients, TB was suspected by radiologists based on the presence of enlarged intrathoracic lymph nodes. In seven cases (24%) patients were referred for a CT scan and these results prompted GHC physicians to suspect TB in four of these cases.

Tuberculin testing was performed in 17 patients whose length of hospital stay was longer than 5 days (59% of all patients) but the result was negative in all cases. Eleven patients (38%) were subjected to commercially available serological tests to detect *M. tuberculosis *antibodies: one case was "positive" and "negative" results in the remaining ten cases were used as a further (incorrect) proof of rejection of TB as a possible diagnosis.

In all 29 cases a sputum sample was microscopically examined for the presence of acid-fast bacilli by the GHC laboratories according to national standards of hospital care for newly admitted HIV-positive patients. However, only in three patients (10%) was more than one sputum sample taken despite current national and international recommendations [14-16]. Microscopy results were negative in all cases. No culture examination for TB or general microbiology was performed.

Overall despite the apparent negative results, in one third of the cases (n = 9) GHC physicians believed that TB was a differential diagnosis. HIV consultants and TB physicians were invited to review 19 (66%) patients; however they had rejected a possible diagnosis of tuberculosis and insisted on further investigation arguing that the symptoms were explained by other accompanying conditions.

All admitted patients received broad spectrum antibacterial therapy that included co-trimoxazole, cephalosporins and erythromycin. None were given HAART or anti-tuberculous therapy; unfortunately without performing a CD4 the usual trigger for ARV therapy would not have been met.

The autopsy results confirmed that in 16 cases (55%) *M. tuberculosis *was both extrapulmonary as well as pulmonary; in 5 patients (17%) in addition to pulmonary TB, TB meningitis was diagnosed (Table 2).

**Table 2:** Autopsy results (n = 29)

*Lung and intrathoracic lymph nodes TB lesions (N, %)*	*Additional TB lesions detected in (n, %):*			*TB sepsis* *(N, %)*
		
	Meninges	Endocardium	Liver and kidneys	
26 (90%)	7 (24%)	1 (3%)	15 (52%)	3 (10%)

In over half of the patients (n = 17; 59%) other opportunistic infections were diagnosed (Table 3).

**Table 3:** Opportunistic infections diagnosed post-mortem in HIV-patients died from TB (n = 29)

*Illness*	*Frequency*	
	
	N	%
Candidiasis	7	24
Herpes	6	21
PCP	1	3
Rotavirus infection	1	3
CMV	1	3

PCP - pneumocyctic pneumonia.

CMV - cytomegalovirus

## Discussion

In middle- and high incidence TB countries this disease is one of the commonest opportunistic infections affecting HIV-positive people causing the majority of AIDS-associated death. Altered immune response leads to atypical clinical presentation of TB resulting into miss-diagnosis and an extremely rapid progression to death [17-19].

The initiation of the study was triggered by lead TB physicians in Togliatti who were concerned that active TB was not recognized in deceased patients. In all study cases, known risk factors for TB were reflected in the medical charts but were not taken into account by treating physicians. Negative tuberculin tests, atypical radiological presentation, and non-specific symptoms did not exclude TB in HIV-positive patients but simply reflected severe immunosupression and should have prompted tuberculosis physicians to consider it as a possible diagnosis.

Unfortunately essential tests for CD4 count were performed in few patients only despite their wide availability; ironically almost all patients were tested for the presence of mycobacterial antibodies, i.e. largely worthless tests that are not recommended by national and international guidelines due to their very low specificity. A recently issued policy statement by the World Health Organisation advises strongly against the use of commercial serological assays presenting evidence for their inconsistent and imprecise findings [20]. Unfortunately the negative results of the serological tests were interpreted as proof against TB as a differential diagnosis. However, the very short time between admission and death in several cases might have limited the chances of hospital physicians to perform the full range of diagnostic tests. The very severe clinical presentation of these patients might have influenced treating physicians to choose a palliative care approach and not to pursue aggressive diagnostic and treatment strategies. Unfortunately, due to the very limited routine clinical data available we can only hypothesise rather than make conclusive statement.

Invited TB consultants and HIV physicians rejected the possibility of TB despite radiological reports suggesting TB as a possible diagnosis. Unfortunately only microscopy was performed on patients' sputum - test of a low sensitivity especially in the setting of GHC institutions not specializing in tuberculosis diagnosis; rapid molecular screening tests and bacteriological culture which would have been very helpful and are available in the city were not performed. As a result, all patients remained at GHC hospitals without being referred to specialized TB units, and received neither anti-tuberculosis treatment nor HAART. Remaining in general wards of the GHC hospitals, these patients were potential sources of infection for other patients. Although drug susceptibility testing was not available for these patients, extremely high rates of drug resistance including MDR have been reported in this region [21,22] and the deceased patients could have been infected with resistant strains further exacerbating infection control problems.

None of the patients received HAART prior to admission and physicians at the AIDS center stated that the reason for this was the assumption of a high probability of non-adherence among those patients; a limited availability of HAART would presumably have influenced the decision not to initiate treatment. In addition many of these patients (n = 20, 69%) failed to present at regular check-ups making it difficult for physicians to initiate and maintain therapy. These findings do not necessarily reflect inadequate care by AIDS physicians but rather point to a strong need to improve social support and adherence of marginalized HIV-infected individuals including treatment of alcohol and drug-abuse.

## Limitations

The study has several limitations. Due to its retrospective nature, gathered information could only reflect the data available from medical charts and autopsy reports which were often incomplete and did not necessarily contain the desired level of detail. However, this analysis is the first attempt by tuberculosis physicians at the level of a large Russian city to openly identify several major gaps in the work of TB, AIDS and general care physicians. The use of autopsy-proven TB cases gave a clear point of reference for an analysis of diagnostic errors and patients' death clearly suggested the potential for diagnostic delay and poor management. The findings are particularly relevant for Togliatti as one of the cities most affected by the HIV-epidemic in Russia. Moreover, for a city with considerable wealth and a developed medical infrastructure, one might assume the availability of a high level of medical expertise but the study suggested the opposite.

## Conclusions

Intensive training of physicians in diagnostics and management of AIDS and AIDS-associated illnesses and improvement of their understanding of the benefits and limitations of laboratory diagnosis is vital and has now taken place as a result of this study. It is important to increase awareness of TB amongst general physicians and that TB can occur at any stage of HIV-infection as well as its frequent atypical presentation in severely immunosuppressed individuals. When TB is suspected, two to three sputum samples should be examined both microscopically and using a liquid (and solid) culture method while anti-TB treatment should be initiated promptly without awaiting for the bacteriological results. Introduction of rapid molecular diagnostics for TB and drug resistance is essential in these settings. Similarly early and appropriate initiation of HAART therapy is essential. Social support and treatment of accompanying alcohol- and drug-abuse are necessary to ensure better adherence of marginalized HIV-infected individuals to diagnostic procedures and treatment.

## Competing interests

The authors declare that they have no competing interests.

## Authors' contributions

FD. Designed the study: YB, VT, IT, SM, NM, LF, SK, IF. Analysed the data: YB, VT, IT, FD. Collected data: VT, IT, SM, NM, LF, SK, IF. Wrote the first draft of the paper: YB, FD. Contributed to the writing of the paper: VT, IT, SM, NM, LF, SK, IF. Agree with manuscript's results and conclusions: YB, VT, IT, SM, NM, LF, SK, IF. All authors read and approved the final manuscript.

## Pre-publication history

The pre-publication history for this paper can be accessed here:

http://www.biomedcentral.com/1471-2334/11/276/prepub
